# A new electoral bottom-up model of institutional governance

**DOI:** 10.1038/s41598-025-87322-y

**Published:** 2025-01-31

**Authors:** Carlos M. Garrido, Francisco C. Santos, Elias Fernández Domingos, Ana M. Nunes, Jorge M. Pacheco

**Affiliations:** 1 BioSystems and Integrative Sciences Institute, Faculdade de Ciências da Universidade de Lisboa Campo Grande, 1749-016 Lisbon, Portugal; 2 ATP-group, 2744-016 Porto Salvo, Portugal; 3 INESC-ID, Universidade de Lisboa, 2744-016 Porto Salvo, Portugal; 4 Instituto Superior Técnico, Universidade de Lisboa, 2744-016 Porto Salvo, Portugal; 5 AI Lab, Computer Science Department, Vrije Universiteit Brussel, Pleinlaan 9, 3rd Floor, 1050 Brussels, Belgium; 6 MLG, Département D’Informatique, Université Libre de Bruxelles, Boulevard Du Triomphe, CP 212, 1050 Brussels, Belgium; 7 Departamento de Física, Faculdade de Ciências da Universidade de Lisboa, Campo Grande, 1749-016 Lisboa, Portugal

**Keywords:** Cooperation, Collective risk dilemma, Evolutionary game theory, Stochastic processes, Climate sciences, Ecology, Applied mathematics

## Abstract

The sustainable governance of Global Risky Commons (GRC)—global commons in the presence of a sizable risk of overall failure—is ubiquitous and requires a global solution. A prominent example is the mitigation of the adverse effects of global warming. In this context, the Collective Risk Dilemma (CRD) provides a convenient baseline model which captures many important features associated with GRC type problems by formulating them as problems of cooperation. Here we make use of the CRD to develop, for the first time, a bottom-up institutional governance framework of GRC. We find that the endogenous creation of local institutions that require a minimum consensus amongst group members—who, in turn, decide the nature of the institution (reward/punishment) via an electoral process—leads to higher overall cooperation than previously proposed designs, especially at low risk, proving that carrots and sticks implemented through local voting processes are more powerful than other designs. The stochastic evolutionary game theoretical model framework developed here further allows us to directly compare our results with those stemming from previous models of institutional governance. The model and the methods employed here are relevant and general enough to be applied to a variety of contemporary interdisciplinary problems.

## Introduction

Global Warming, considered by the United Nations (UN) as one of the most important global problems we face^1^, is a typical example of a GRC whose solution requires worldwide cooperation. The Collective Risk Dilemma (CRD) models theoretically this GRC as a risky, threshold public goods game. It has been widely employed in recent years^2–18^, and represents a theoretical adaptation of the original design employed in behavioral experiments^19–21^. Cooperation in the CRD means paying a cost to mitigate, whose benefits may or may not become available to all. Thus, the temptation to free-ride on the benefits produced by others at their own expense is an inescapable component of the model. However, in order to secure the provision of the public good, a minimum number of Cooperators is needed, without whom every member of the group is at risk of losing all they have (for details, see Methods).

Previous work^7^ making use of the CRD indicates that sanctioning institutions help to solve the Global Problem provided they are endogenously created at a Local scale, whereas global institutions (such as the UN) do not change the qualitative behavior of the CRD as a function of risk compared to a scenario where no institutions are at work (black solid line in Fig. 3), supporting the widely repeated motto “think globally, act locally”.

**Figure 1:**
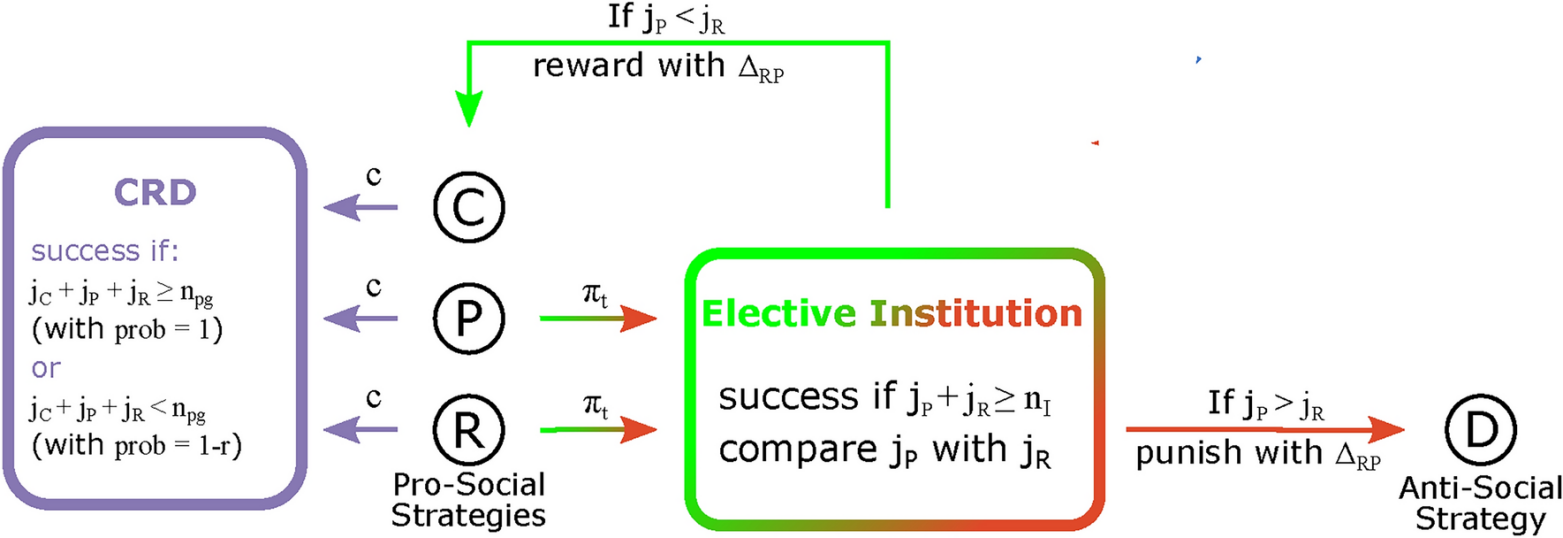
Schematic representation of the Bottom-Up institutional model developed here to address the sustainable governance of GRC making use of the CRD dilemma. There are three pro-social strategies: C (Cooperators) P (Punishers) and R (rewarders) that contribute with a fraction *c* of their endowment to the CRD (left-pointing arrows); and one anti-social strategy, D (Defectors), that does not contribute. P and R also pay an additional tax $\pi _t$ to fund an Electoral institution (right-pointing arrows) which decides, based on a majority rule, how to use its revenue $\Delta _{RP}$: Either to Punish the Ds, to reward the pro-social strategies: C, P and R or to reward the pro-social and Punish the anti-social in case of a tie. In the well-mixed approximation, the configuration of the population can be specified by the state vector $\varvec{i}=\{i_C,i_P,i_R,i_D\}$, where $i_S$ is the number of individuals using strategy *S* in the population. We use a corresponding notation to specify the composition of each group: $\varvec{j}=\{j_C,j_P,j_R,j_D\}$ where $j_S$ is the number of individuals using strategy *S* in the group—see Methods for full details of the model.

Furthermore, even though some global agreements, such as the Montreal Protocol^22^ and the Kigali Amendment to the Montreal Protocol^23^, look promising in tackling global dilemmas related to the production of harmful gases, an all-encompassing agreement addressing Global Warming, reached at a global scale, would still require the implementation of solutions adapted to particular specificities and challenges at a local scale, calling for a polycentric approach to best deal with each particular regional challenge^24^. Consequently, we shall concentrate, in the following, on local, endogenously created, institutions.

The creation of institutions poses another costly public good, leading to a second-order social dilemma that separates those who contribute to the institution that provides the incentives from those who do not contribute. Our model not only takes these points into consideration but also lets individuals in each group decide, though a voting process, the nature of the institution they create: Either a sanctioning institution (that provides negative incentives to anti-social group members) or a rewarding institution (that provides positive incentives to pro-social group members).

**Figure 2:**
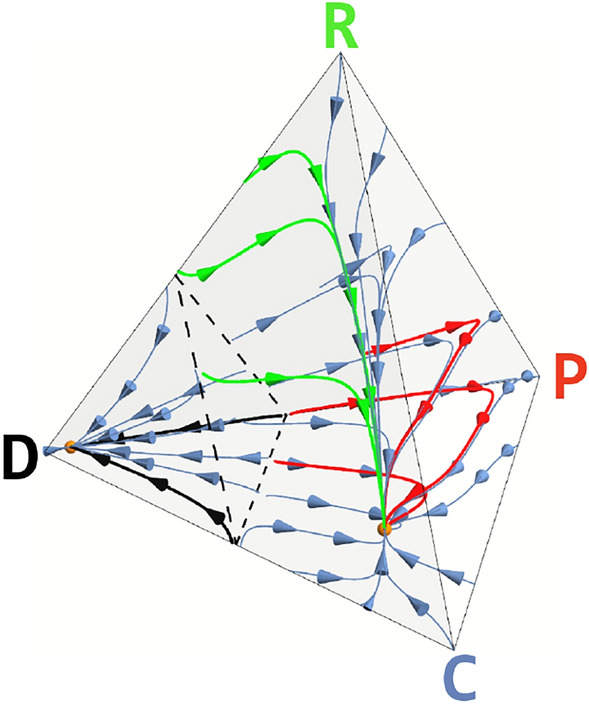
Stochastic Evolutionary dynamics of the Bottom-Up Electoral model developed here, showing that the overall dynamics is dominated, for most parameter values, by 2 interior attractors, both depicted with solid orange spheres: One close to the ALL-D configuration, and another (“cooperative”) at a configuration where Cs clearly dominate. The black and blue arrows (to the left of the dashed triangle) illustrate the most likely paths (in a stochastic sense) that converge to the ALL-D attractor, whereas the blue, green and red arrows (to the right of the dashed triangle) illustrate those paths that converge to the cooperative attractor. Whenever $r < 0.5$ (for the model parameters chosen) the population remains, most of the time, near the ALL-D configuration, well below the fitness barrier illustrated by black dashed lines that qualitatively represent the intersection of the (quasi-planar) fitness barrier top surface with the surface of the simplex. Whenever $r > 0.7$ (see Fig. 3) the population is now able both to tunnel through the barrier and to evolve towards the cooperative attractor where (in a total of 70 individuals) there are 52 Cs, 7 Rs, 6 Ps and 5 Ds. Finally, among the plethora of trajectories (in blue) converging to the “cooperative” attractor, we distinguish 2 types belonging to different “classes” of evolutionary paths: 1) In green we illustrate paths where convergence evolves mostly due to an initial rise of Rs without any significant participation of Ps. 2) In red, paths where convergence evolves mostly through a significant increase of Ps without any significant participation of Rs. As discussed in detail in the main text, the first class of trajectories is more abundant than the second class. Parameters used: $Z=70$ $\mu =1/Z$, $\beta =5$, $N=8$, $n_{pg}=6$, $n_I=2$, $b=1$, $c=0.1$, $\delta =2$, $\pi _t=0.03$, $r=0.8$.

Our new Bottom-Up institutional design is illustrated in Fig. 1. To successfully create an institution, a minimum number ($n_{I}$) of institution creators needs to be present in each group. The nature (reward/punishment) of the Local institution thus created will be decided via a majority rule, that is, an electoral process performed by the institution creators: A majority of rewarders (cf. Fig. 1 for strategy identification) leads to a rewarding institution, whereas a majority of Punishers originates a sanctioning institution. Mathematically, this implies augmenting the CRD to include, besides Cooperators (C) and Defectors (D), rewarders (R) and Punishers (P) (see Methods). Consequently, the evolutionary dynamics of the 4 types of individuals will unfold in a simplex (phase space) with the shape of a regular tetrahedron, as illustrated in Fig. 2. This setting increases considerably the complexity of the present population dynamics compared to previous approaches^2–18^.

In the stochastic population dynamics with mutations considered here (see Methods), the evolutionary dynamics is generally characterized by the occurrence of 2 attractors in the interior of the simplex. Figure 2 illustrates the prevailing scenario (for intermediate to high values of risk *r*, $r \ge 0.4$) where the attractors are depicted with orange solid spheres^25^. These 2 attractors originate 2 basins of attraction that are typically separated by a “fitness barrier” associated with configurations with a nearly constant number of Ds in the population, leading to a planar-like surface whose intersection with the simplex is illustrated (qualitatively) by black dashed lines.

For low risk, and despite the possible occurrence of 2 attractors, the population is not able to tunnel across the fitness barrier, leading to an average configuration of the population that remains very close to the ALL-D configuration.

At higher risk, the population is now able to explore the other side of the fitness barrier, such that the dynamics becomes dominated by the attractor located in the cooperating region of the simplex, at a configuration dominated by Cs (cf. Fig. 2).

In the Supplementary Information (SI) we provide details of the stationary distribution (defined in Methods) associated with these different scenarios and we also test the robustness of the results shown to changes in the model parameters.

**Figure 3:**
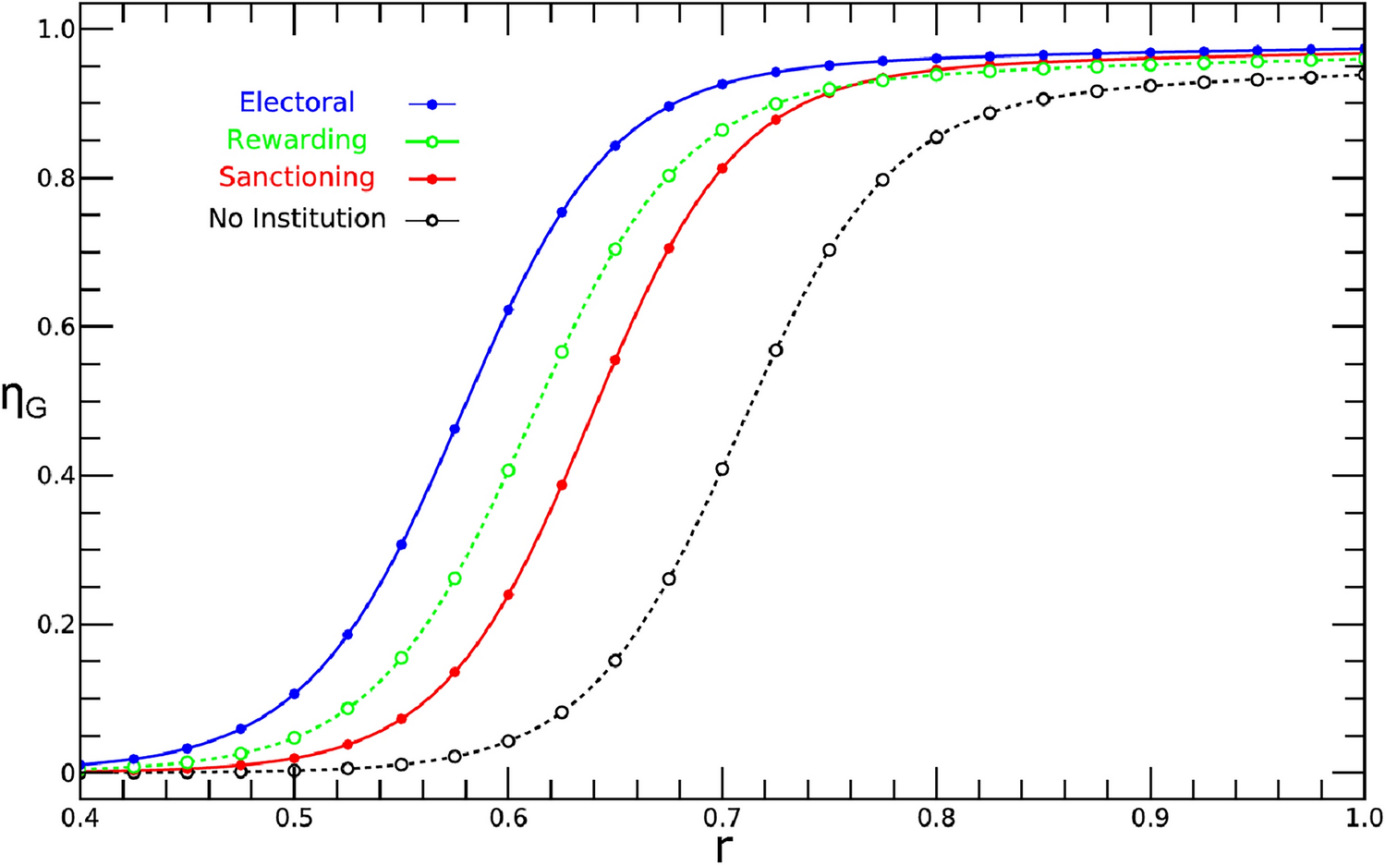
The population average group achievement $\eta _G$ (defined in Methods) is plotted as a function of risk for the case of i) No institutions (black dashed line with open circles); ii) Local sanctioning (punishing) institutions (red solid line with solid circles); iii) Local rewarding institutions (green dashed line with open circles) and iv) Local Electoral institutions (blue solid line with solid circles). Clearly, the present bottom-up approach leads to higher overall cooperation, for all values of risk, compared to other models developed previously. Parameters used: $Z=140$, $\mu =1/Z$, $\beta =2$ $N=8$, $n_{pg}=6$, $n_I=2$, $b=1$, $c=0.1$, $\pi _t=0.03$, $\delta =2$.

A ubiquitous feature of the CRD (also observed in present model, c.f. Fig. 3) is associated with a transition from a defector dominated dynamics at low-risk to a cooperator dominated dynamics at high-risk, irrespective of the existence of institutions, as well as of their nature. This behavior translates into a *S*-shape profile when one plots ${\eta }_G$— the population average group achievement, defined in Methods—as a function of risk. Clearly, as shown previously, different institutional designs typically contribute to change the critical values of risk at which the transition from Defection to Cooperation occurs, as well as the rate of this transition.

The quantity ${\eta }_G$ not only captures these transitions in a single curve, but it has the additional advantage of allowing one to directly compare the performance of models of different inherent dimensionality. These models were generally developed making use of different parametrizations of costs, benefits, rewards and sanctions. Because our model encompasses all mechanisms introduced before, we are able to compare the performance of all models in the present framework. This is precisely what is shown in Fig. 3 where the main findings of this work are shown.

## Results

In the absence of institutions (black dashed line with open circles, where evolutionary dynamics occurs in a one-dimensional simplex^2^) and for the model parameter values indicated in Fig. 3, one needs a critical risk value $r^* \approx 0.755$ to reach ${\eta }_G \approx 0.5$.

Local institutions, either sanctioning (red solid line with solid circles) or rewarding (green dashed line with open circles), whose evolutionary dynamics unfolds in a two-dimensional simplex^7,12,18^) considerably improve the overall prospects of cooperation in the CRD: Now ${\eta }_G \approx 0.5$ is reached at $r^* \approx 0.695$ and $r^* \approx 0.675$, respectively.

Further improvement (blue solid line with solid circles, where evolutionary dynamics unfolds in a three-dimensional simplex—see Fig. 2) is obtained via the present model employing local, electoral institutions, of a nature (reward/punishment) that is decided at a group level, case by case, leading to $r^* \approx 0.640$.

The results in Fig. 3 show that the present model, which encompasses electoral institutions of a dual nature, outperforms previous approaches considering reward-only or punishment-only institutions (see also^18^), more so if we take the rate of change from defection to cooperation, estimated by computing, at the $r^*$ values reported before, the quantity $\left( d {\eta }_G / d r \right) _{r=r^*}$, which is maximal for the present model.

Figure 2 proves helpful in developing an intuition to understand the superior performance of the present model compared to others, in particular to models of reward-only institutions. Indeed, in reward-only models, the evolutionary dynamics proceeds exclusively along the triangular D-C-R face of the tetrahedron. This will constrain possible paths towards the cooperative attractor to those pertaining to the green class illustrated in Fig. 2. The paths belonging to the red class, also illustrated in Fig. 2 to be accessible in the present Electoral model, are not available in reward-only models, which explains the less cooperative dynamics observed for these models. A similar argument is valid for punishment-only institutions which, per se (and for most model parameters), lead to poorer overall performance compared to reward-only institutions. Indeed, if we estimate the fraction of red-type to green-type evolutionary trajectories obtained via computer simulations of time evolution, starting from the vicinity of the ALL-D configuration, we obtain a (risk-dependent) value of $\sim 0.2$ (for $r=0.8$), which supports the intuition that punishment-only institutions are less efficient than reward-only institutions, although their combination, via the electoral process developed here, provides superior results. Similar conclusions can be drawn if we compute the fraction of time punishment institutions prevail over rewarding institutions and vice-versa, as shown in the SI.

It is also worth pointing out that, as illustrated in Fig. 2, Cs clearly dominate in the configuration defined by the cooperative attractor of the stochastic evolutionary dynamics; however, it is important to realize that there are small, but non-negligible, numbers of both Rs and Ps present in the population in this configuration. These numbers (note that, for the parameters employed in Fig. 2 the average number of Ps and Rs grow from 1 at $r=0.5$ to 5 and 6 at $r=0.8$, respectively) ensure the existence of a reservoir of institutional individuals that are able to maintain some policing of groups, rewarding/sanctioning their members accordingly.

## Discussion

As stated in the beginning, sanctions are harder to implement than rewards, even when they result from the celebration of International Agreements. Therefore, it is gratifying to realize that rewarding institutions alone, which have an easier job in implementing their goals, are generally more efficient to promote cooperation than sanctioning institutions, cf. Fig. 3.

The present model, however, reinforces the idea that sanctioning institutions are important in “policing” free-riders^7,12,26^, here at the group level, where sanctioning is more likely to be effective.

In the present model, our analysis concentrated on the long time distribution of strategies in the population, by computing the stationary distribution and associated observables. This methodology allowed us to compare the performance of different rewarding and sanctioning models with different inherent dimensionality, which was our primary goal. This said, we did not address in detail other aspects of the present model which are also of relevance, namely the behavior of strategies in time. As is well known, evolutionary game models may lead to oscillatory behaviour, both under deterministic^27–29^ and stochastic^30,31^ dynamics. In the present model, the prevailing scenario portrayed in Fig. 2 does not lead to periodic oscillations, although one cannot rule out the occurrence of oscillatory behavior for particular parameter combinations or in situations where spatial effects are added to the model.

To summarize, we developed a new bottom-up model of institutional governance of risky commons which allows the creation of flexible, local institutions, by introducing a threshold requirement for their formation at a local, group level, and whose nature (reward or punishment) is decided, also locally, via a voting process (majority rule).

This model not only promotes the self-organization of cooperation in the population for values of risk significantly lower than previous models, but also keeps reward as the dominant mechanism while keeping sanctioning mechanisms available at all times, and applied at a local level (that is, in smaller groups), which helps to make their efficiency more feasible.

## Methods

In all cases, we shall employ evolutionary game theory of finite populations, where the (stochastic) dynamics proceeds in discrete time through a sequence of one-step selection-mutation processes, with mutation probability $\mu$ and selection pressure $\beta$ (see below).

We further assume a well-mixed population of size *Z* where individuals form groups of size *N* and engage in a CRD with group threshold $n_{pg}$. Institutions are endogenously created via the contributions from pro-social individuals in each group (except Cooperators), a process that requires a minimum threshold $n_I$ of those individuals present in each group.

Individuals adopt, each, one of the 4 following strategies: Cooperators (Cs), Punishers (Ps), rewarders (Rs) and Defectors (Ds).

Each possible configuration of the population can be represented by a state vector $\varvec{i}=\{i_C,i_P,i_R,i_D\}$, where $i_S$ is the number of individuals using strategy *S* in the population.

Individuals have an initial endowment of *b* and meet in groups of size *N*, whose composition can be specified by a corresponding state vector $\varvec{j}=\{j_C,j_P,j_R,j_D\}$ where $j_S$ represents the number of individuals using strategy *S* in the group.

Three strategies—C, R and P are pro-social, and thus individuals who employ them contribute a fraction *c* of their endowment *b* to the CRD. D, in turn, is an anti-social strategy, as individuals choose not to contribute and thus keep all of their initial endowment—see Fig. 1.

For the CRD to succeed in producing a public good, the number of pro-social individuals in a group needs to exceed a group threshold $n_{pg}$.

Whenever the threshold is not met, then everyone in the group will lose their endowments with a risk probability *r*. Therefore the base payoff related to the CRD can be written

1 $$\begin{aligned} \pi _0(j) = b \cdot \theta \left( j_{CPR};n_{pg}\right) + (1-r)b \cdot \bar{\theta }\left( j_{CPR};n_{pg}\right) \end{aligned}$$

where $\theta (x; y)$ is the Heavide step function ($\theta (x; y)=1$ if $x \ge y$ and 0 otherwise), $\bar{\theta }=1-\theta$ and $j_{CPR}=j_{C}+j_{P}+j_{R}$.

Ps and Rs contribute an additional tax, $\pi _t$, to a local electoral institution at the group level. This institution will be created and will have an impact proportional to the total contributions of pro-social individuals provided a minimum threshold of participants $n_I$ is reached in a group of size *N*

2 $$\begin{aligned} \Delta _{RP} (j_P, j_R) = \delta \cdot \pi _t \cdot j_{PR} \cdot \theta \left( j_{PR};n_I\right) \end{aligned}$$

where $\delta \ge 1$ is a multiplication factor that reflects a possible return on the amount contributed via taxes to the formation of an institution.

Each local institution is here an electoral institution because the decision on the nature of the institution is based on a majority rule:

If the number of Ps is larger than the number of Rs then all anti-social individuals (Ds) in the group will lose some amount (same to all) totaling $\Delta _{RP}$; if the number of Ps is smaller than the number of Rs then $\Delta _{RP}$ will be evenly distributed by all the pro-social individuals (Cs, Ps and Rs); if a tie is reached, i.e. the number of Ps is the same as Rs, then $\Delta _{RP}/2$ is used to reward pro-social individuals and the other $\Delta _{RP}/2$ is used to punish anti-social individuals. Figure 1 illustrates the workings of the present model.

The resulting payoffs associated with each strategy read,

3a $$\begin{aligned} \pi _C \left( \varvec{j}\right)&= \pi _0(j) -cb + \frac{\Delta _{RP} (j_P, j_R)}{j_{CPR}}\theta _{el}\left( j_R; j_P\right) \end{aligned}$$

3b $$\begin{aligned} \pi _P \left( \varvec{j}\right)&= \pi _C \left( \varvec{j}\right) - \pi _t \end{aligned}$$

3c $$\begin{aligned} \pi _R \left( \varvec{j}\right)&= \pi _P \left( \varvec{j}\right) \end{aligned}$$

3d $$\begin{aligned} \pi _D = \left( \varvec{j}\right)&\pi _0(j) - \frac{\Delta _{RP} (j_P, j_R)}{N-j_{CPR}}\theta _{el}\left( j_P; j_R\right) \end{aligned}$$

where $\theta _{el}(x;y)$ is a threshold function which encodes the elective institution described above

4 $$\begin{aligned} \theta _{el}(x; y) = {\left\{ \begin{array}{ll} 0 & if \;x<y\\ 1/2 & if \; x=y\\ 1 & otherwise \end{array}\right. } \end{aligned}$$

**Stochastic Evolutionary Dynamics**

In the framework of Evolutionary Game Theory of finite, well-mixed populations, the fitness $f_{S_k}(\varvec{i})$ of an individual adopting strategy $S_k$ in a population with a configuration $\varvec{i}$ is given by the average payoff of strategy $S_k$ for that particular configuration, given by

5 $$\begin{aligned} f_{S_k}(\varvec{i}) = \left( {\begin{array}{c}Z-1\\ N-1\end{array}}\right) ^{-1} \; \sum _{\begin{array}{c} \boldsymbol{j}=0\\ j_{DCPR}=N-1 \end{array}}^{\boldsymbol{j}=N-1} \pi _{S_k}\left( \varvec{j}\right) \left( {\begin{array}{c}i_{S_k}-1\\ j_{S_k}\end{array}}\right) \prod _{\begin{array}{c} l=1\\ l\ne k \end{array}}^{4}\left( {\begin{array}{c}i_{S_l}\\ j_{S_l}\end{array}}\right) \end{aligned}$$

where a hypergeometric sampling of the population is performed, $j_{DCPR}=j_{D}+j_{C}+j_{P}+j_{R}$ and $\varvec{j}=A$ stands for $j_{D}=j_{C}=j_{P}=j_{R}=A$.

The evolution of the population takes place via a series of discrete one-step selection-mutation processes; as a result, at any discrete time-step, the state of the population depends only on its present configuration, which means that its evolution can be described by a Markov Process, whose probability density function (PDF) $p_i$ satisfies the discrete Master Equation^32^

6 $$\begin{aligned} p_{\varvec{i}}(t+\tau )-p_{\varvec{i}}(t)= \sum _{\varvec{i'}} \left\{ T_{\varvec{i}\varvec{i'}} p_{\varvec{i}}(t)-T_{\varvec{i'}\varvec{i}} p_{\varvec{i}}(t) \right\} \end{aligned}$$

where $T_{\varvec{i}\varvec{i'}}$ and $T_{\varvec{i'}\varvec{i}}$ are the transition probabilities between states $\varvec{i}$ and $\varvec{i'}$ and vice-versa respectively.

The non-diagonal transition probabilities can be readily calculated given the discrete one-step nature of the selection-mutation process,

7 $$\begin{aligned} T_{S_l\rightarrow S_k}(\varvec{i}) = (1-\mu )\frac{i_l}{Z}\frac{i_k}{Z-1} P\left( f_{S_k}-f_{S_l}\right) + \mu \frac{i_l}{(S-1)Z} \end{aligned}$$

where $\mu$ is the mutation probability and the notation $T_{S_l\rightarrow S_k}(\varvec{i})$ refers to the transition probability starting from state $\varvec{i}$ and having one individual with strategy $S_l$ change its strategy to strategy $S_k$ via the pairwise comparison rule^33,34^ $P(x)=[1+exp\left( -\beta x \right) ]^{-1}$ which employs the Fermi function from statistical physics, where the inverse temperature $\beta$ ($\beta \in \mathbb {R}^+_0$) represents here the strength of natural selection. The diagonal transition probabilities can be calculated using the relation $T_{\varvec{ii}}=1-\sum _{\varvec{i'}\ne \varvec{i}}T_{\varvec{i'i}}$.

We shall be interested in determining the stationary distribution $\left( \overline{p_{\varvec{i}}}\right)$ which is given by^8^ the eigenvector associated with the highest eigenvalue (of value 1) for the transition matrix $\textbf{T}^T$: $\overline{p_i}(\varvec{i})=\left[ T_{\varvec{i}\varvec{i'}}\right] ^T \overline{p_i}(\varvec{i})$.

The transition probabilities further allow us to compute the most likely path the population will follow starting from a given configuration, by means of the gradient of selection $\nabla _{\varvec{i}}$

8 $$\begin{aligned} \nabla _{\varvec{i}} = \sum _{k=1}^s \left( T_{\varvec{i}}^{S_k +}-T_{\varvec{i}}^{S_k -}\right) \varvec{u}_k \end{aligned}$$

where $T_{\varvec{i}}^{S_k +}$ and $T_{\varvec{i}}^{S_k -}$ represent the probability the number of individuals adopting strategy $S_k$ increases or decreases by one, respectively and the unit vectors $\varvec{u}_k$ define the basis of the phase space dynamics.

Finally, making use of the stationary distribution $\overline{p_i}$ we can compute other quantities of interest such as ${\eta }_G$, the population average group achievement,

9 $$\begin{aligned} {\eta }_G =\sum _{\varvec{i}} \overline{p_i}(\varvec{i}) a_G(\varvec{i}) \end{aligned}$$

where $a_G(\varvec{i})$ is the so-called group achievement, computed for each population configuration $\varvec{i}$ by averaging over all possible group configurations for which success in the CRD is granted

10 $$\begin{aligned} a_G(\varvec{i})=\left( {\begin{array}{c}Z\\ N\end{array}}\right) ^{-1} \sum _{\begin{array}{c} \boldsymbol{j}=0\\ j_{DCPR}=N \end{array}}^{\boldsymbol{j}=N} \theta \left( j_{CPR};n_{pg}\right) \prod _{l=1}^{4}\left( {\begin{array}{c}i_{S_l}\\ j_{S_l}\end{array}}\right) \end{aligned}$$

Naturally, we may construct similar formulas to compute the incidence of reward or punishment for each configuration $\varvec{i}$ and subsequently compute the population average amount of reward and punishment for each set of model parameters—see SI. A freely available implementation of the general equations above can be found in the repository^35^. For a detailed acccount of the methods employed see^36^.

## Supplementary Information

Supplementary Information.

## Data Availability

All data supporting the findings of this study are available in the Plots presented within the paper and its Supplementary Information. Files with raw data used to generate the plots are available from the corresponding author on reasonable request.
