# Supplementary material for: A new electoral bottom-up model of institutional governance
Source: Sci Rep. 2025 Jan 31;15:3865. doi: 10.1038/s41598-025-87322-y (PMC11785748; doi:10.1038/s41598-025-87322-y)
Supplement: Supplementary file 1 — Supplementary Information. [file 41598_2025_87322_MOESM1_ESM.pdf]

A New Electoral Bottom-Up Model of Institutional Governance

Carlos M. Garrido[1, 2], **Francisco C. Santos**[3, 4, 2], **Elias Fernández Domingos**[5, 6], **Ana M. Nunes**[1, 7], **and Jorge M. Pacheco**[3, 2]

[1]BioSystems and Integrative Sciences Institute, Faculdade de Ciências da Universidade de Lisboa Campo Grande, 1749-016 Lisboa, Portugal
[2]ATP-group, P-2744-016 Porto Salvo, Portugal
[3]INESC-ID, Universidade de Lisboa, 2744-016 Porto Salvo, Portugal
[4]Instituto Superior Técnico, Universidade de Lisboa, 2744-016 Porto Salvo, Portugal
[5]AI lab, Computer Science Department, Vrije Universiteit Brussel, Pleinlaan 9, 3rd Floor, 1050 Brussels, Belgium
[6]MLG, Département D'Informatique, Université Libre de Bruxelles, Boulevard Du Triomphe, CP 212, 1050 Brussels, Belgium
[7]Departamento de Física, Faculdade de Ciências da Universidade de Lisboa Campo Grande, 1749-016 Lisboa, Portugal

Supplementary Information

In the following, we explore the impact of changing the different model parameters. The results are quite intuitive, in the sense that they correspond to what one expects from general threshold public goods games:
Increasing population size Z and selection pressure β will act to sharpen the transition from defection to cooperation as a function of risk, as shown in **SI** Figs. 1 and 2, respectively. Correspondingly, larger group sizes N will require a higher risk for the same transition to occur, as shown in **SI** Fig. 3, the same happening if, as shown in **SI** Fig.4, one increases the public good threshold n_{pg}. Finally, in what concerns the institution parameters (n_I,π_t and δ) we show in **SI** Fig. 5 how increasing the institutional threshold n_I acts to increase the risk at which the transition from defection to cooperation takes place. This transition, in turn, is affected in different ways as we change the individual taxes π_t incurred by both $\boldsymbol{P}$s and $\boldsymbol{R}$s, as shown in **SI** Fig. 6 (maintaining all other model parameters invariant). Indeed, for very small values of π_t, small tax increases will be beneficial to overall cooperation. Further increases, however, inevitably become detrimental to overall cooperation. Note, however, that high values of π_t are generally beneficial to cooperation at low-risk. Finally, as shown in **SI** Fig. 7, increasing the institutional return δ helps cooperation to emerge at a lower risk.

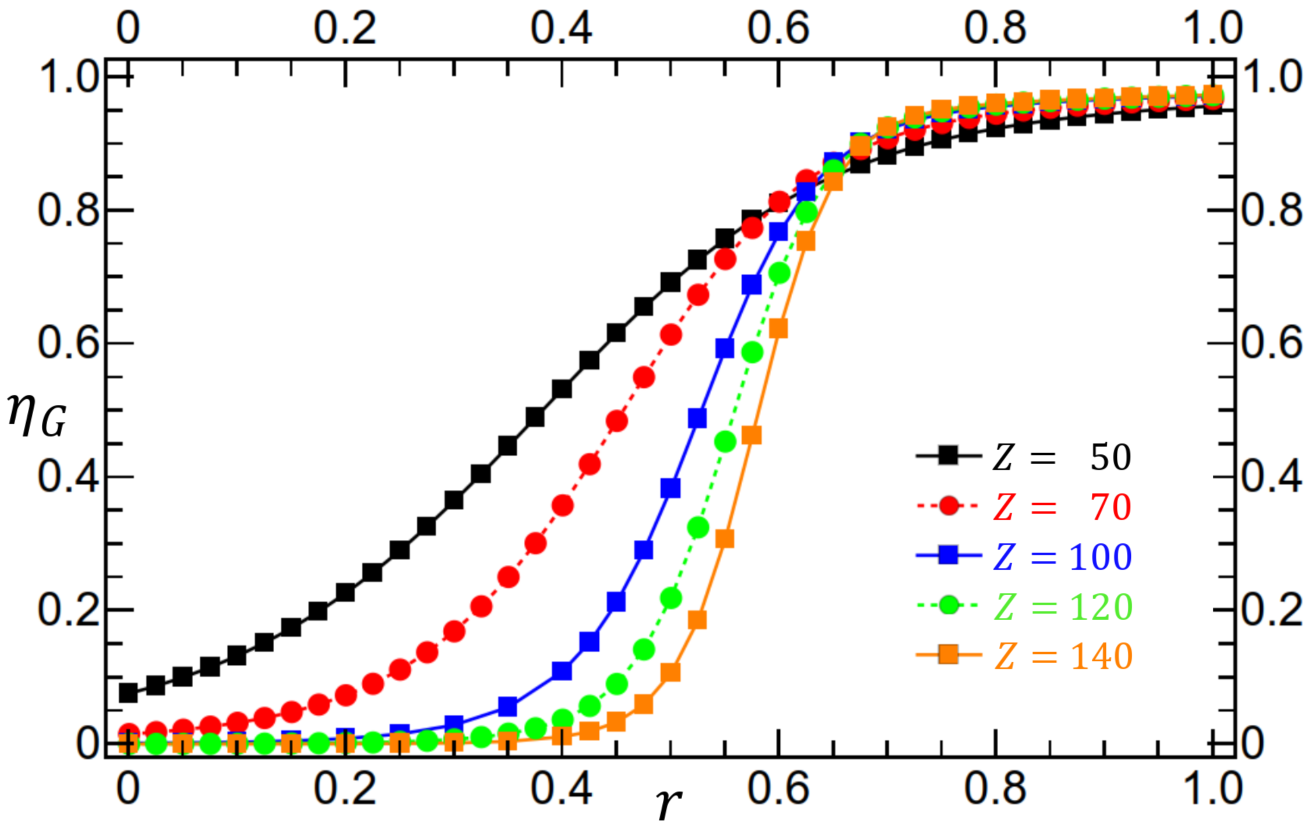

Figure 1. η_G **versus risk for different population sizes**. We changed population size Z maintaining all other model parameters constant (see below). With increasing Z one observes that the transition from defection ($\eta_G \to 0$) to cooperation ($\eta_G \to 1$) occurs in an increasingly narrower interval of risk. At the same time, the value of risk at which $\eta_G = 0.5$ increases. In the following figures, we shall use the value $Z = 100$ as the reference population size. As the figure shows, this value shows an intermediate behavior between small and very large population sizes, retaining the computational feasibility of the calculations, which become prohibitevely expensive for large populations. Parameter values used: $b = 1$, $c = 0.1$, $\mu = 1/Z$, $\beta = 2$ $N = 8$, $n_{pg} = 6$, $n_I = 2$, $\pi_t = 0.03$, $\delta = 2$.

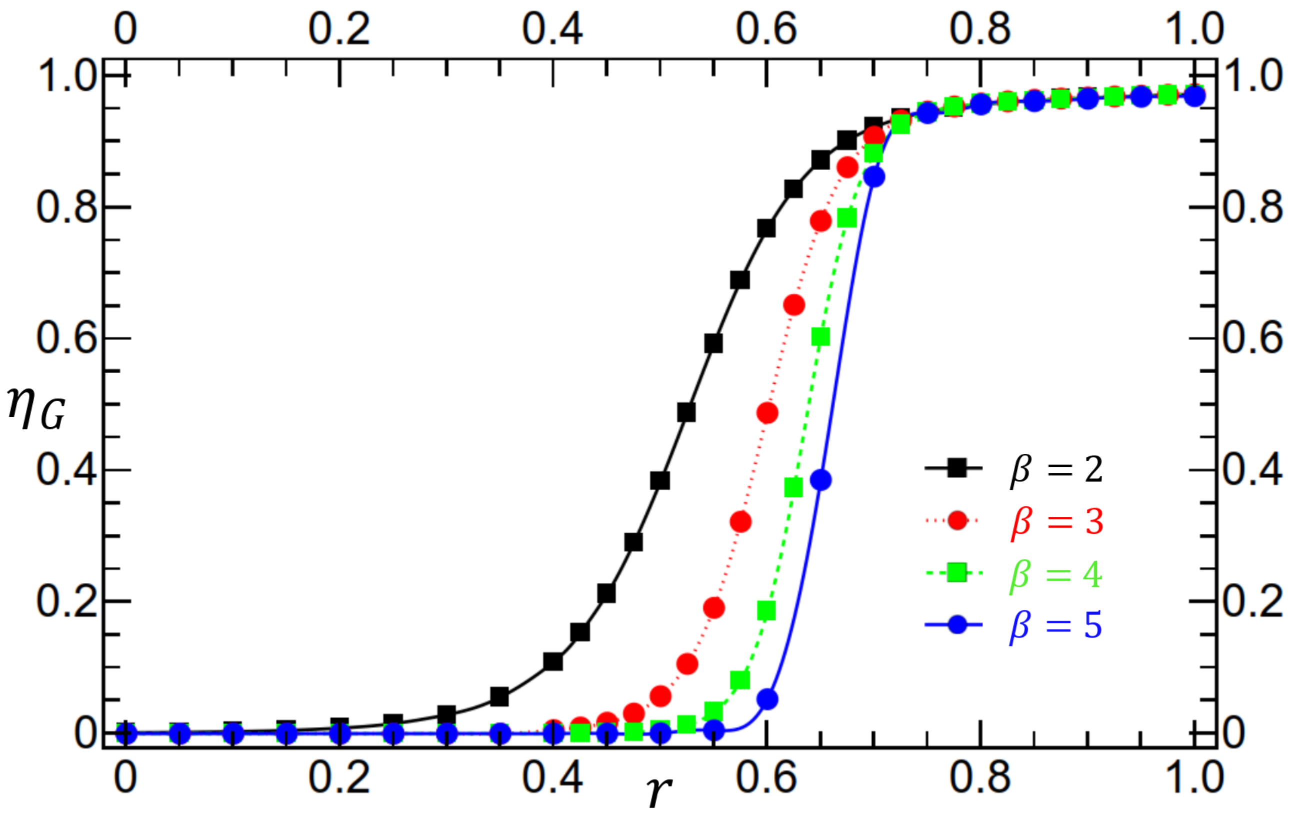

Figure 2. **η_G versus risk for different values of the selection pressure β.** We changed the selection pressure β while maintaining all other model parameters constant (see below). Similar to changing population size Z, increasing β sharpens the transition from defection ($\eta_G \to 0$) to cooperation ($\eta_G \to 1$), also shifting to higher values the value of risk at which $\eta_G = 0.5$. Parameter values used: $b = 1$, $c = 0.1$, $Z = 100$, $\mu = 1/Z$, $\beta = 2$ $N = 8$, $n_{pg} = 6$, $n_I = 2$, $\pi_t = 0.03$, $\delta = 2$.

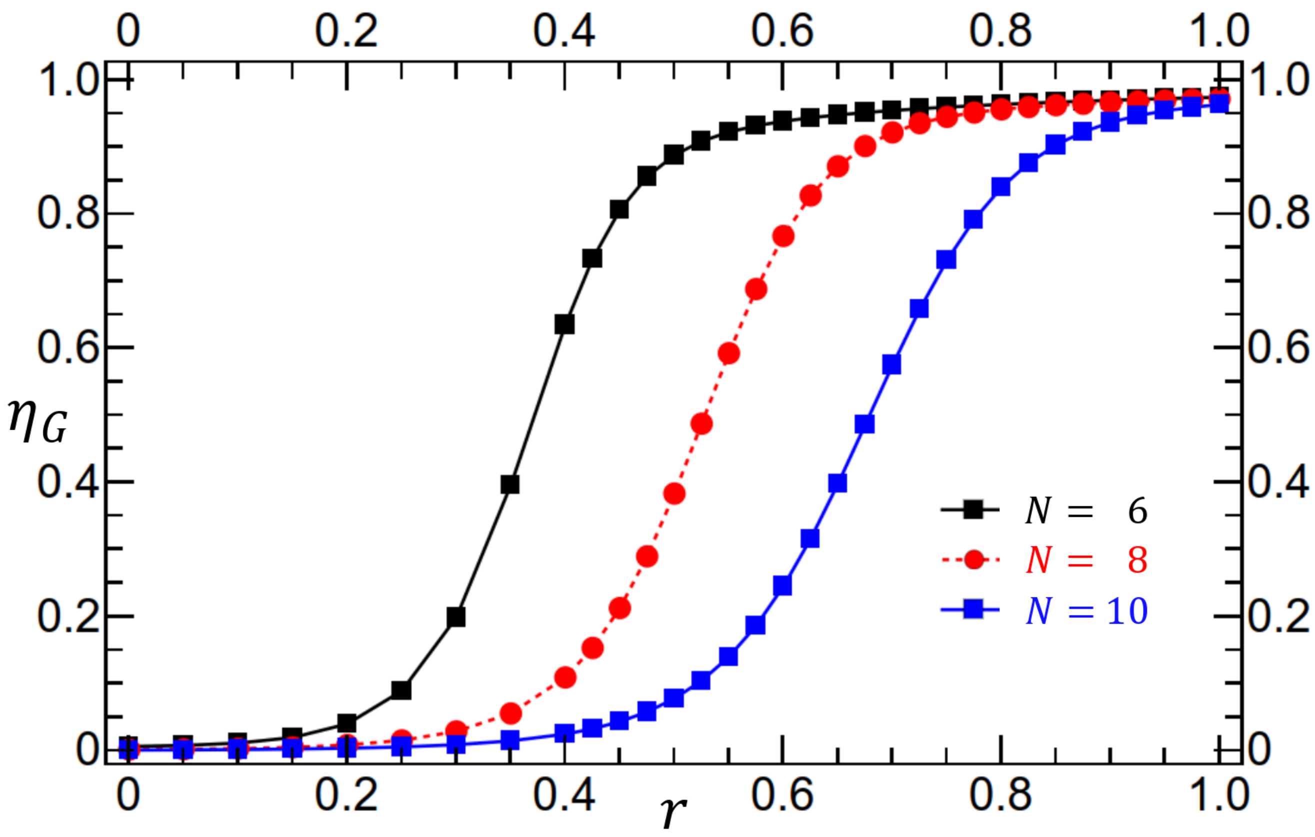

Figure 3. η_G **versus risk for different values of the group size** N. We changed group size N maintaining all other model parameters constant except n_{pg} (see below), which we also changed to keep the ratio n_{pg}/N in a range of comparative values. This means that we use $n_{pg} = 4$ for $N = 6$ ($n_{pg}/N = 0.67$), $n_{pg} = 6$ for $N = 8$ ($n_{pg}/N = 0.75$) and $n_{pg} = 8$ for $N = 10$ ($n_{pg}/N = 0.80$). With increasing N (and, correspondingly, n_{pg}) one observes that the transition from defection ($\eta_G \to 0$) to cooperation ($\eta_G \to 1$) occurs at increasingly higher values of risk. This is a general feature of threshold Public Goods Games that is retained in the present model.
Parameter values used: $b = 1$, $c = 0.1$, $Z = 100$, $\mu = 1/Z$, $\beta = 2$, $n_I = 2$, $\pi_t = 0.03$, $\delta = 2$.

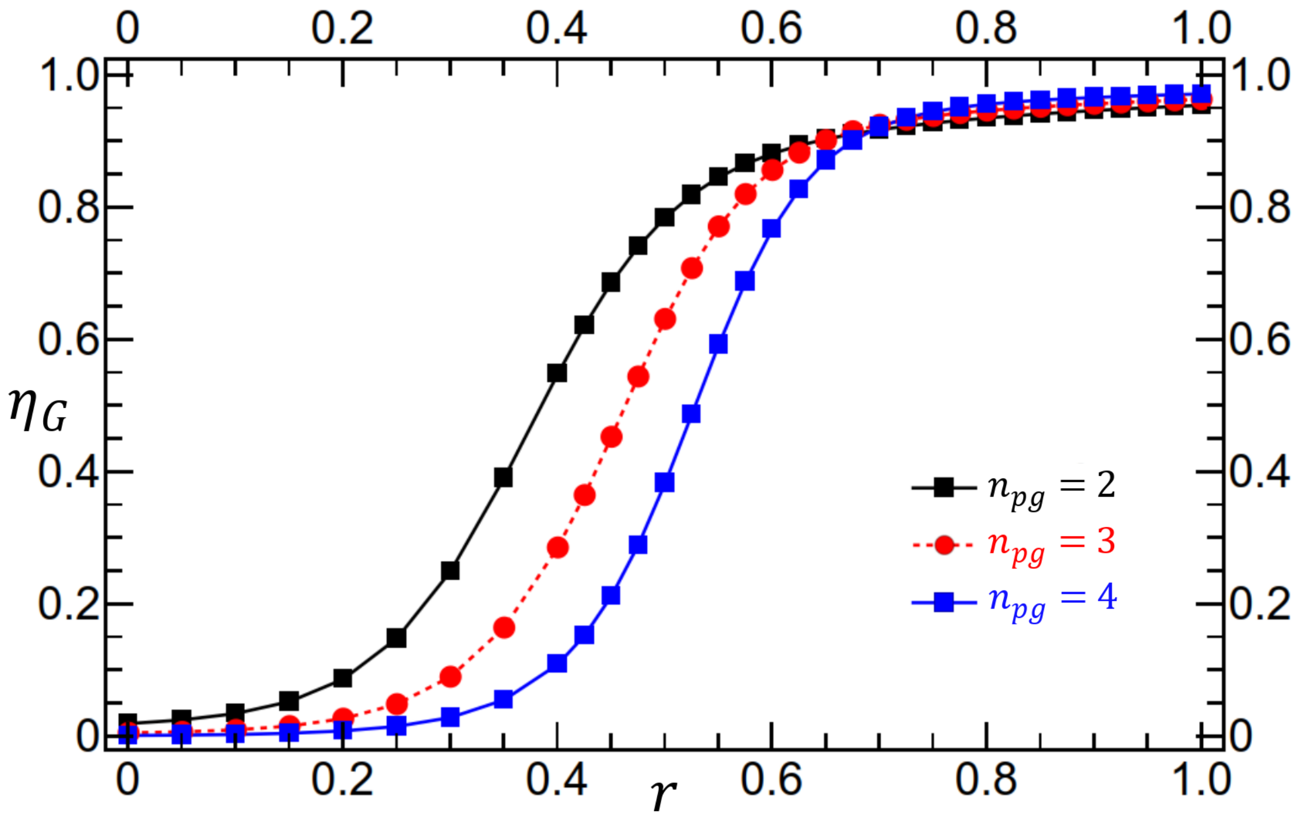

Figure 4. **η_G versus risk for different group threshold values**. We changed the group threshold value n_{pg} maintaining all other model parameters constant (see below). With increasing n_{pg} one observes that the transition from defection ($\eta_G \to 0$) to cooperation ($\eta_G \to 1$) occurs at increasingly higher values of risk. This is a general feature of threshold Public Goods Games that is retained in the present model.
Parameter values used: $b = 1$, $c = 0.1$, $Z = 100$, $\mu = 1/Z$, $\beta = 2$ $N = 8$, $n_{pg} = 6$, $n_I = 2$, $\pi_t = 0.03$, $\delta = 2$.

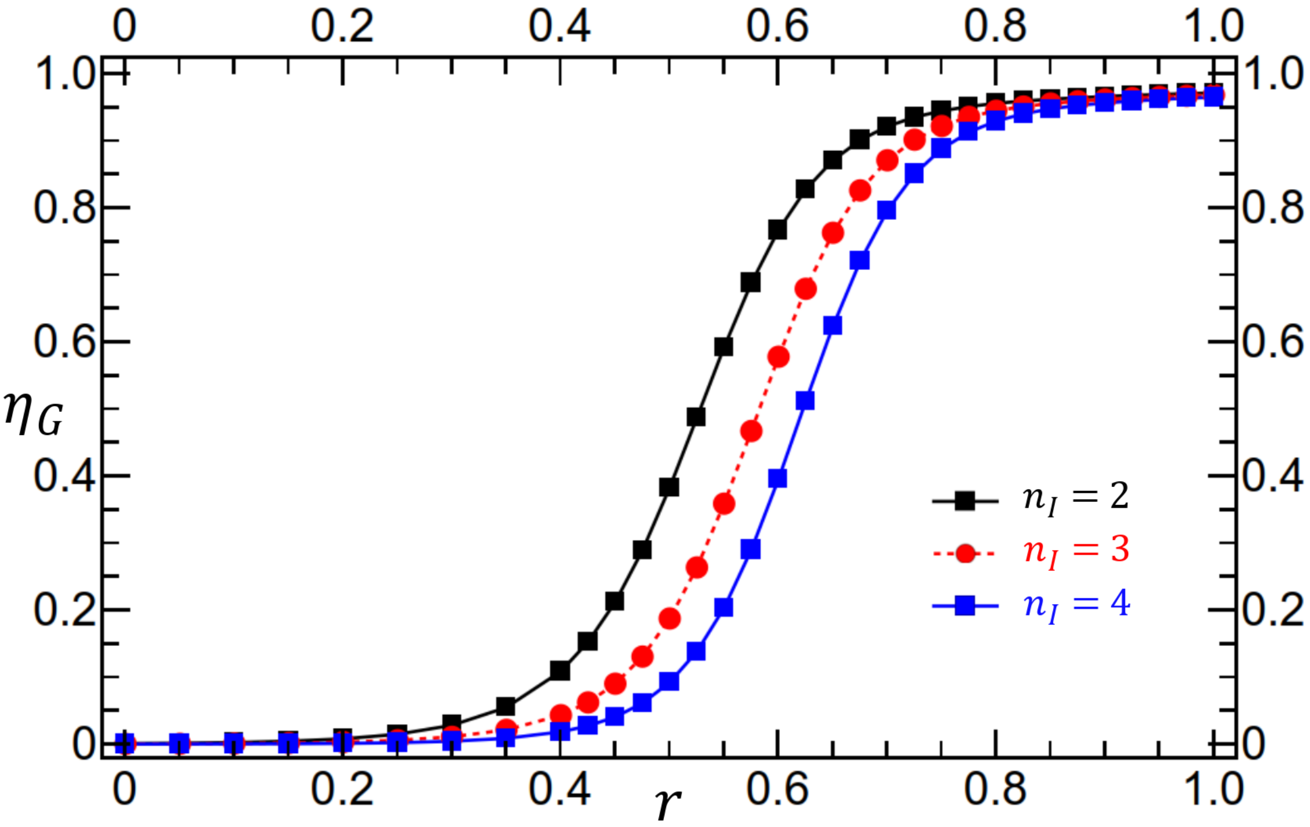

Figure 5. η_G **versus risk for different institutional threshold sizes**. We changed institutional threshold (n_I) value maintaining all other model parameters constant (see below). With increasing n_I one observes that the transition from defection ($\eta_G \to 0$) to cooperation ($\eta_G \to 1$) occurs at increasingly higher values of risk, as one would expect.
Parameter values used: $b = 1$, $c = 0.1$, $Z = 100$, $\mu = 1/Z$, $\beta = 2$ $N = 8$, $n_{pg} = 6$, $\pi_t = 0.03$, $\delta = 2$.

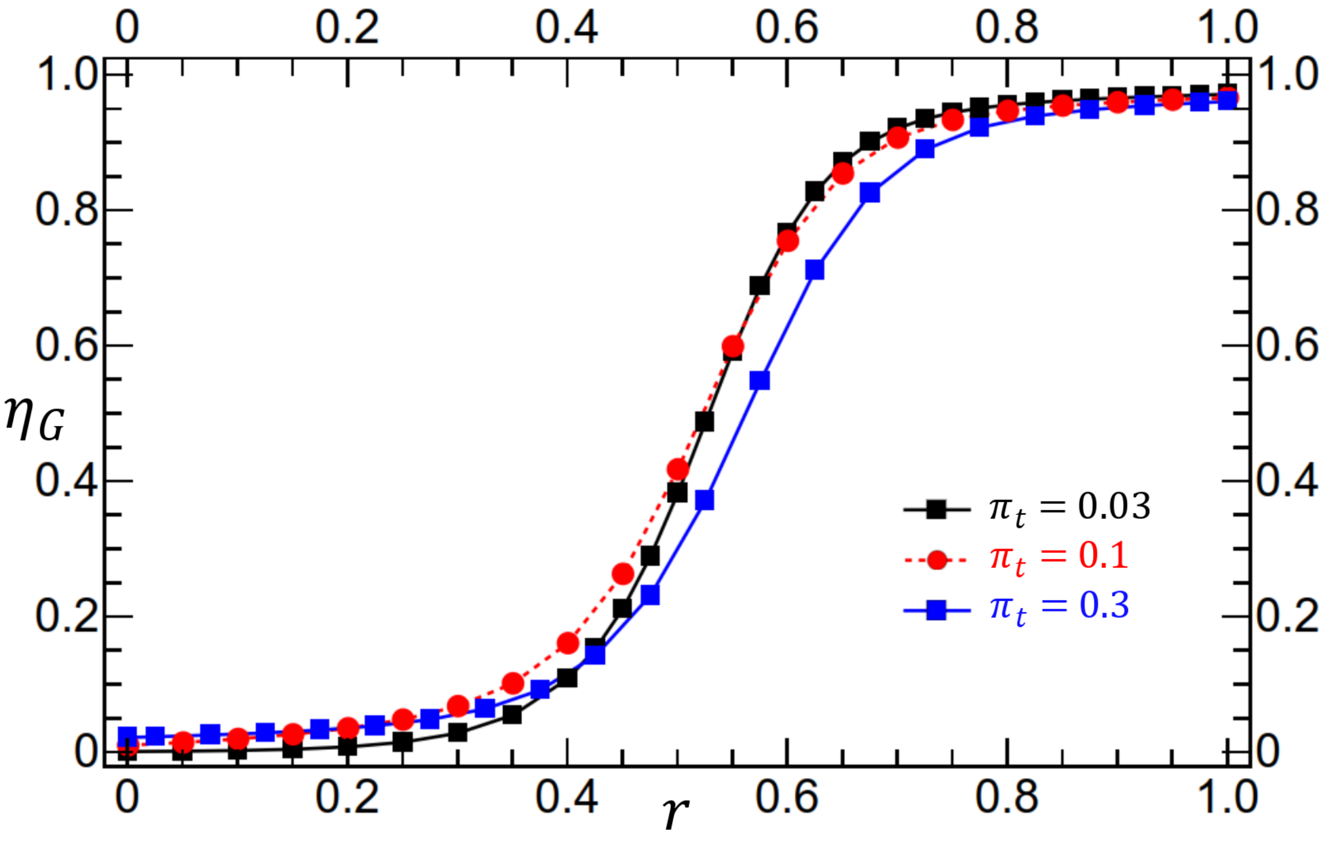

Figure 6. η_G **versus risk for different individual institutional tax values**. We changed the amount that pro-institutional individuals (***P***s and ***R***s) must contribute to create an institution at the group level (π_t). We maintained all other model parameters constant (see below). The lowest value plotted ($\pi_t = 0.03$) corresponds to the most used value throughout the manuscript. For smaller values of π_t (not shown) the transition from defection ($\eta_G \to 0$) to cooperation ($\eta_G \to 1$) occurs for slightly larger values of risk compared to $\pi_t = 0.03$. In other words, increasing π_t is beneficial to cooperation for low values of π_t. With increasing π_t we still observe an overall benefit regarding the emergence of cooperation (see the results for $\pi_t = 0.1$), but this benefit disappears when the amount contributed via π_t becomes too large, as shown in the figure for an order of magnitude increase of π_t. This said, increasing taxes is beneficial at very low risk, being detrimental at high risk. Parameter values used: $b = 1$, $c = 0.1$, $Z = 100$, $\mu = 1/Z$, $\beta = 2$ $N = 8$, $n_{pg} = 6$, $n_I = 2$, $\delta = 2$.

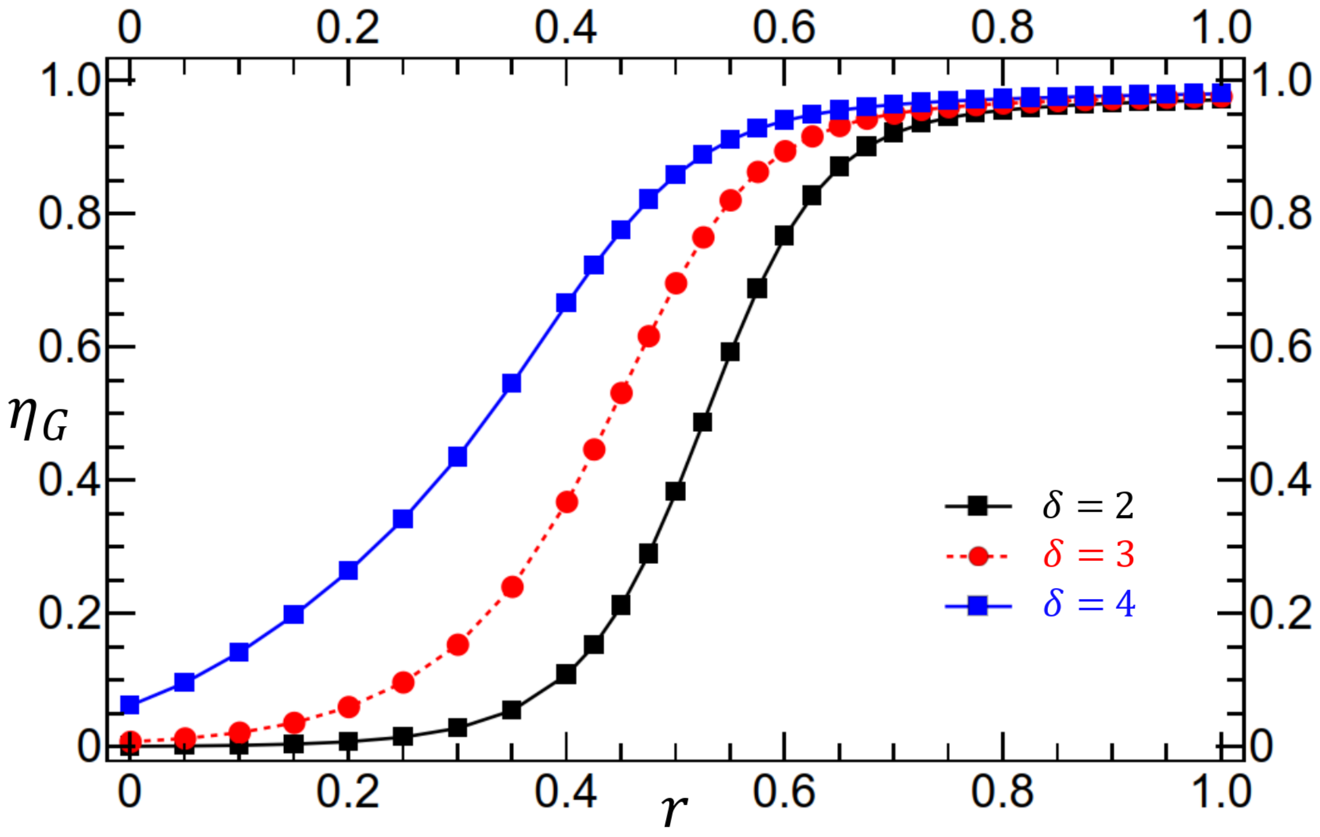

Figure 7. η_G **versus risk for different institutional return values** δ. We changed the value of the institutional return δ maintaining all other model parameters constant (see below). With increasing δ one observes that, as expected the positive role of institutions is greatly enhanced, sizably reducing the risk value at which $\eta_G = 0.5$ occurs.
Parameter values used: $Z = 100$, $\mu = 1/Z$, $\beta = 2$ $N = 8$, $n_I = 2$, $b = 1$, $c = 0.1$, $\pi_t = 0.03$, $\delta = 2$.

Stationary Distributions

In **SI** Fig. 8 we plot the stationary distribution associated with a particular example, at high risk ($r = 0.7$) where overall cooperation dominates the dynamics. For $S = 4$ strategies, the visualization of the stationary distribution is non-trivial, and therefore we divide the figure in three panels, plotting the stationary distribution in domains of increasingly lower dimensionality.

In panel **a)** we show the full simplex, choosing a perspective that best illustrates the accumulation of strength in the vicinity of the **D-C-R** plane, although it is clear that, in the vicinity of the cooperative attractor, the stationary distribution extends somewhat inside the simplex, towards the "P"-vertex. This becomes clearly visible if we make a triangular cut of the simplex at a constant value of number of **D**s in the population, in this case $i_D = 9$ (the number of **D**s at which the maximum of the stationary distribution occurs). The result is shown in panel **b)**, for the triangle joining the configurations $\mathbf{R}^* \rightarrow i = (09,00,00,61)$, $\mathbf{C}^* \rightarrow i = (09,61,00,00)$ and $\mathbf{P}^* \rightarrow i = (09,00,61,00)$, also illustrated in panel **a)** by means of a blue triangle, showing how strength penetrates into the interior of the simplex. Finally, panel **c)** shows the relative intensity of the two attractors referred in the main text – the cooperative attractor at high number of **C**s and the defective attractor at high number of **D**s. We thus plot the values of the stationary distribution along the edge **D-C**, illustrating the fact that the population spends more time in the vicinity of the cooperative attractor than in the vicinity of the other attractor.

SI Fig. 9 shows, for the parameters indicated, all configurations with a probability of reward (in green) and punishment (in red) greater than 90%; This three-dimensional distribution encompasses the **P-R** edge, along which we plot the probability distributions to punish (in red) to reward (in green) as well as to punish-and-reward (in black) along this edge.
Note that, similarly to what was done when computing η_G, we can also investigate the behavior of the population average probabilities of reward and punishment. Indeed, we can compute the population average probabilities

$$\mathbf{Prob}(P) = \sum_i \overline{p_i}(i) a_P(i) \tag{1a}$$

$$\mathbf{Prob}(R) = \sum_i \overline{p_i}(i) a_R(i) \tag{1b}$$

where

$$a_P(i) = \sum_{(j:j_k=0)}^{(j:j_k=N)} \theta\left(j_{PR}; n_I\right) \cdot \theta\left(P; R\right) \cdot H(j; i, Z, N) \tag{2a}$$

$$a_R(i) = \sum_{(j:j_k=0)}^{(j:j_k=N)} \theta\left(j_{PR}; n_I\right) \cdot \theta\left(R; P\right) \cdot H(j; i, Z, N) \tag{2b}$$

In **SI** Fig. 10 we plot $\mathbf{Prob}(R)$, $\mathbf{Prob}(P)$ and the ratio $\mathbf{Prob}(P)/\mathbf{Prob}(R)$ as a function of risk. Clearly, the reward probability is always larger than punishment, and they both grow with increasing risk. However, as risk increases, the ratio decreases and stabilizes as η_G approaches 1. The figure clearly shows that, for any value of risk, both reward and punishment are important and contribute to the success of the electoral model.

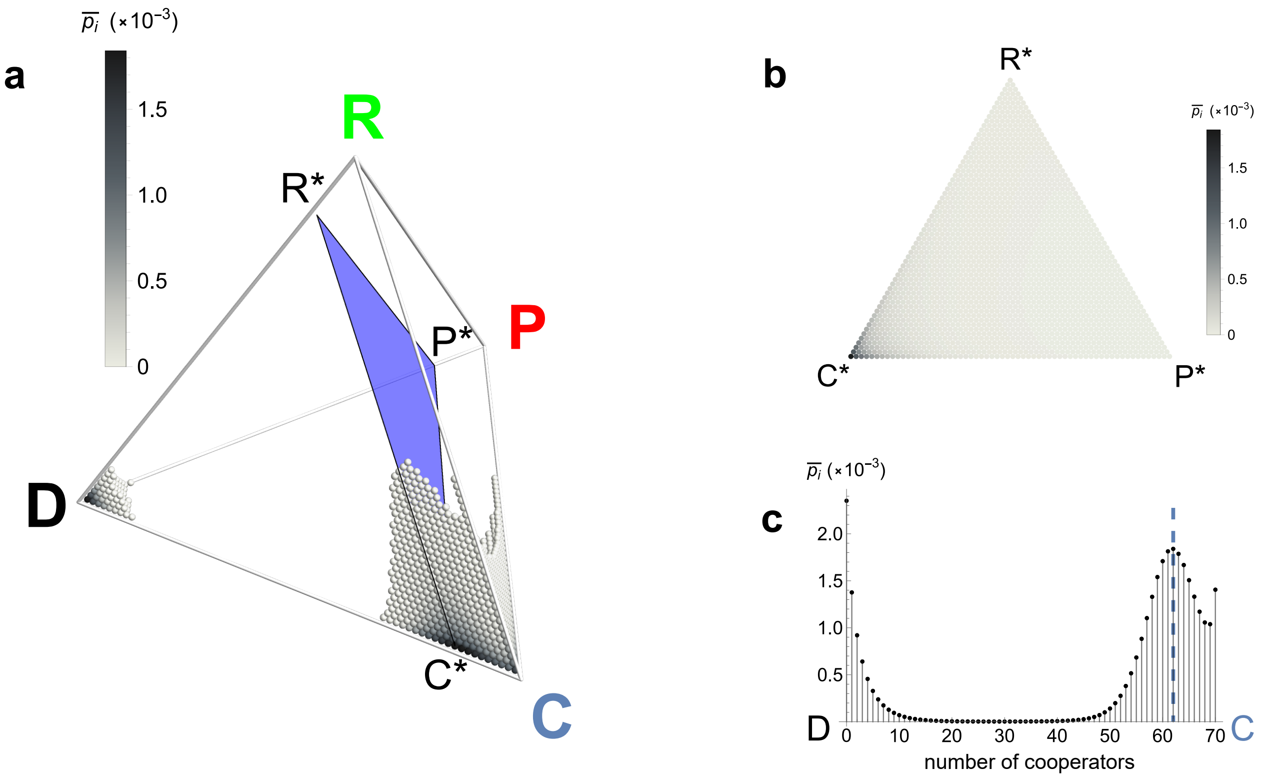

Figure 8. Stationary distribution for a specific set of model parameters. We plot the stationary distribution $\overline{p_i}$ (see Methods in main text for details) for: **a)** All possible configurations associated with a finite population of size $Z = 70$ (see model parameters at the end). Since we selected $r = 0.7$, the cooperative attractor associated with a high number of **C**s dominates the stochastic evolutionary dynamics, indicating that the population spends more time in its vicinity. Furthermore, the picture also illustrates the fact that the stationary distribution is mostly concentrated on the **D-C-R** planar face of the tetrahedron, although it is also apparent that it extends towards the interior of the simplex. Panel **b)** illustrates the extent to which the stationary distribution penetrates into the interior of the simplex, by defining the blue triangle shown in panel *a* that joins the configurations $R^* \to i = (08, 00, 00, 62)$, $C^* \to i = (09, 62, 00, 00)$ and $P^* \to i = (09, 00, 62, 00)$, characterized by, in all cases, the number of **D**s remaining constant and equal to 9, precisely the value at which the stationary distribution is maximal in the vicinity of the cooperative attractor.
Finally, panel **c)** shows the stationary distribution along the edge "D-C", illustrating the fact that the population spends more time in the vicinity of the cooperative attractor than in the vicinity of the defective attractor.
Parameter values used: $b = 1$, $c = 0.1$, $r = 0.7$, $Z = 70$, $\mu = 1/Z$, $\beta = 5$ $N = 8$, $n_{pg} = 6$, $n_I = 2$, $\pi_t = 0.03$, $\delta = 2$.

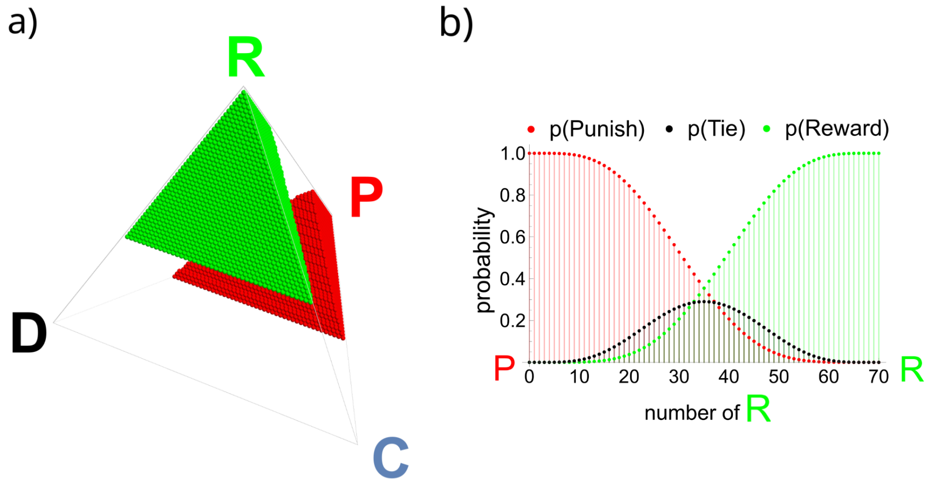

Figure 9. Probability of each incentive type for a specific set of model parameters. We plot: **a)** All the configurations with a probability of reward (in green) and punishment (in red) higher than 90% associated with a finite population of size $Z = 70$ (see model parameters at end). Panel **b)** shows the probability that each type of incentive will be applied as a function of population configuration along the **P-R** edge. p(Tie") represents the probability that a tie happens in a group (j_P=j_R) for a given configuration of the population.
Parameter values used: $Z = 70$, $N = 8$, $n_{pg} = 6$, $n_I = 2$.

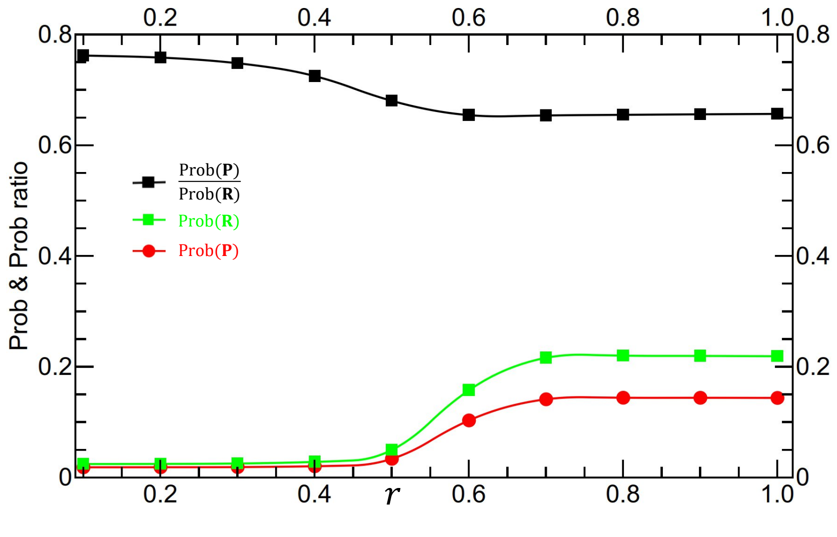

Figure 10. Probability of reward, punishment and their ratio as a function of risk. We plot the probability of punishment (red line with solid circles), of reward (green line with solid squares) as well as the ratio between these two probabilities (black line with solid squares) as a function of risk. Both **Prob**(R) and **Prob**(R) exhibit a similar behavior with risk, stabilizing at high risk, when η_G approaches 1. However, for all values of risk we have that **Prob**(R) > **Prob**(P), although the ratio decreases with increasing risk. Same parameters as in Figure 3 of main text.
